# Non-acute effects of passive heating interventions on cardiometabolic risk and vascular health: systematic review and meta-analysis of randomized controlled trials

**DOI:** 10.1016/j.ajpc.2025.101082

**Published:** 2025-08-17

**Authors:** Rikuta Hamaya, Yuki Joyama, Tomohiro Miyata, Shun-ichiro Fuse, Naho Yamane, Natsuki Maruyama, Hirofumi Kanazawa, Koki Morishita, Howard D. Sesso

**Affiliations:** a Division of Preventive Medicine, Brigham and Women’s Hospital, Boston, MA, USA; b Department of Biostatistics, Columbia University Mailman School of Public Health, NY, NY, USA; c Department of Cardiology, Biwako Ohashi Hospital, Otsu, Shiga, Japan; d School of Medicine, Hokkaido University, Sapporo, Japan; e Department of Medicine, Sado General Hospital, Niigata, Japan; f Department of Epidemiology, Harvard T.H. Chan School of Public Health, Boston, MA, USA; g Department of Paediatrics, Nagano Children’s Hospital, Nagano, Japan; h Department of Anesthesiology, School of Medicine, University of Minnesota, Minneapolis, MN, USA; i Department of Cardiology, Teine Keijinkai Hospital, Sapporo, Hokkaido, Japan

**Keywords:** Passive heating, Meta-analysis, Randomized controlled trial, Blood pressure, Prevention

## Abstract

**Backgrounds:**

Passive heating practices, such as hot water immersion and Finnish sauna bathing, have been associated with reduced cardiovascular disease (CVD) risk in observational studies. This systematic review and meta-analysis aimed to comprehensively evaluate the present literature about non-acute effects of passive heating interventions on cardiometabolic and vascular health in randomized controlled trials (RCTs).

**Methods and Results:**

A comprehensive search was conducted in PubMed, Embase, and Cochrane Central Register of Controlled Trials up to November 4, 2024. We included RCTs that assessed the effects of passive heating interventions of at least one week duration on cardiometabolic and vascular outcomes in adults. Twenty RCTs that tested interventions such as hot water bathing, saunas, hot yoga, and local heating were included in the present review, with durations ranging from 2 to 15 weeks. Our analysis found no significant pooled effects for majority of outcomes, including flow-mediated dilation, pulse wave velocity, resting heart rate, heart rate variability, fasting glucose, HbA1c, total/HDL/LDL cholesterol, triglycerides, and C-reactive protein. For systolic blood pressure (SBP), the overall pooled estimate was not statistically significant (–2.46 mmHg [95 % CI: 5.02 to 0.10]; I²=60.3 %). However, subgroup analyses indicated a significant SBP reduction was confined to systemic (whole-body) heating interventions (–4.11 mmHg [–7.36, –0.86]) and in populations with underlying coronary risk or CVD (–2.52 mmHg [–4.26, –0.79]).

**Conclusions:**

Current evidence from RCTs indicates that passive heating interventions may not improve most of the cardiometabolic or vascular health markers. While a potential reduction in SBP was observed with systemic heating and among adults with coronary risk, this finding should be interpreted with caution due to high heterogeneity and limitations in the included studies.

This systematic review is registered at PROSPERO (registration number: CRD42024621600).

## Highlights

• Passive heating, such as hot water bathing and sauna, has been suggested to have cardioprotectic roles in observational studies.
• In the systematic review of RCTs investigating the effect of passive heating intervention on cardiometabolic risk and vascular health biomarkers, a potential reduction in SBP was observed with systemic heating and among adults with underlying coronary risk or CVD.
• However, the review is limited by the individual study limitations and significant between-study heterogeneity.

## Graphical abstract

**Figure:**
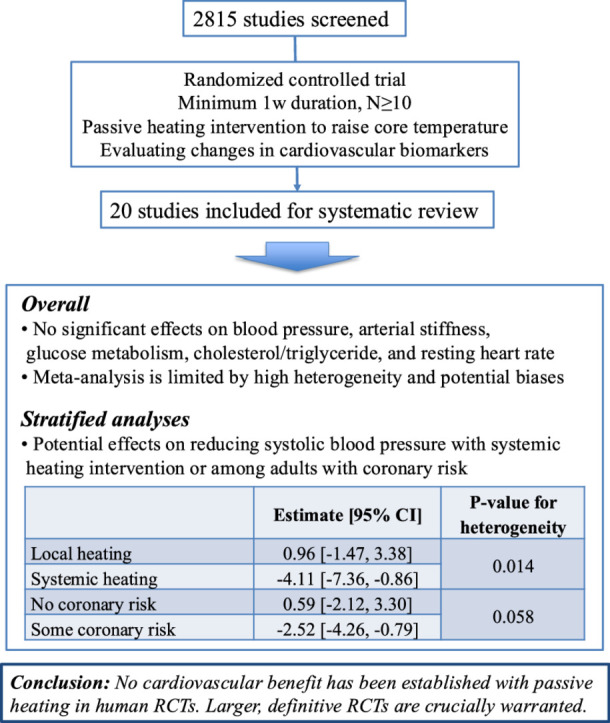

## 1 Introduction

For cardiovascular disease (CVD) prevention, multiple factors have been extensively investigated either in cohort studies or clinical trials, in particular for diet, exercise, drugs, and dietary supplements. However, implementation of CVD primary prevention remains challenging, due to the complex physiological mechanisms and difficulties in modifying unhealthy behaviors. Meanwhile, there are some under-evaluated lifestyle factors that can contribute to CVD prevention common in certain parts of the world. Passive heating is an activity which is popular as hot water immersion or bathing in a bathtub in Japan and as sauna bathing in Finland. Large cohort studies in each country have suggested a striking benefit of passive heating in preventing CVD [1,2], although these studies may not indicate causality [3,4] due to confounding and other potential limitations.

Potential cardio-protective mechanisms of passive heating include improvements in cardiac and vascular function and reductions in oxidative stress, inflammation, and blood pressure [5]. These mechanisms are likely apparent after 1–2 weeks of passive heating, along with other acute physiological changes [5]. There have been several randomized controlled trials (RCTs) to date, with a previous meta-analysis suggesting some benefits of passive heating in improving cardiometabolic risk. Systolic and diastolic blood pressure (SBP and DBP) were reduced by 4 mmHg by the passive heating intervention with high heterogeneity between studies [6]. There were no effects on glucose metabolism and lipids in patients with type 2 diabetes [7]. Notably, sources of heterogeneity across previous RCTs have not been explored, including trial design, type of intervention, or study population.

In addition, studies have investigated the non-acute impacts of passive heating on vascular health such as arterial stiffness and autonomic nerve activities. A 2021 meta-analysis of 5 studies including non-RCTs showed a significant effect of passive heating in improving flow-mediated dilatation (FMD) by 1.95 %, with no effect on resting heart rate (HR) [6]. However, heterogeneity was high and evidence not definitive. Recently, Debray et al. reported an RCT of 8-week Finnish sauna bathing intervention in adults with coronary artery disease, showing no effect in FMD and pulse wave velocity (PWV) [8]. As with cardioprotective mechanisms, the type or regimen of passive heating that may improve vascular functions or affect autonomic nerve activities remains unclear.

To date, no systematic review has investigated non-acute effects of passive heating interventions on comprehensive cardiometabolic and vascular health and explored potential sources of the heterogeneity. Therefore, we systematically reviewed the evidence of passive heating interventions with minimum 1 week of duration for modulations of comprehensive cardiometabolic and vascular health, including BP, glucose metabolism, lipids, and inflammation, arterial stiffness, and measures of autonomic nerve activities. This threshold was chosen to distinguish studies investigating potential non-acute heating adaptations relevant to CVD prevention from those assessing only the immediate physiological responses to a single heat exposure.

## 2 Methods

### 2.1 Patient and public involvement section

Patient and Public were not involved in this study.

### 2.2 Literature selection criteria

We searched publications from PubMed, Embase, and Cochrane Central Register of Controlled Trials from inception until November 4, 2024, focusing on papers written in English or Japanese. We focused on RCTs that investigated the non-acute effects of any type of passive heating intervention, including dry sauna, Finnish sauna, hot yoga, tub bathing and other form of balneology using warm or hot water, and any local heating interventions that aim to raise core temperature, on cardiometabolic risk. Original studies with the following information were included: (1) treatment arms including any types of passive heating interventions that raise core temperature; (2) outcomes including at least one of the following cardiometabolic measurements: SBP and DBP, glucose metabolism (fasting glucose, insulin, or HbA1c), lipids (total/HDL/LDL cholesterol or triglyceride), and inflammatory biomarkers (C-reactive protein [CRP], interleukin-6 [IL-6], and tumor necrosis factor-alpha [TNF-α]); (3) effect estimates of absolute changes; and (4) minimum intervention duration of 1 week; and (5) any type of RCT necessitating active and control arms. The target trial population was adults aged ≥18y without acute illness requiring hospitalization. We excluded trials with sample size <10 (to minimize small-study effects), those with interventions that did not seek to raise core temperature, and those targeting patients with active inflammation (e.g. rheumatoid arthritis) when they evaluated the effect of passive heating on inflammation. For passive heating interventions integrated with exercise, the control arm needed to be exercise alone to evaluate the standalone effect of passive heating. The study design has been registered at PROSPERO (registration number: CRD42024621600). The protocol of this study followed the Preferred Reporting Items for Systemic review and Meta-Analysis (PRISMA) Protocols. This study was exempt from institution review board review as it is a systematic review and meta-analysis of existing published data.

### 2.3 Literature search and data extraction

**Fig. 1:**
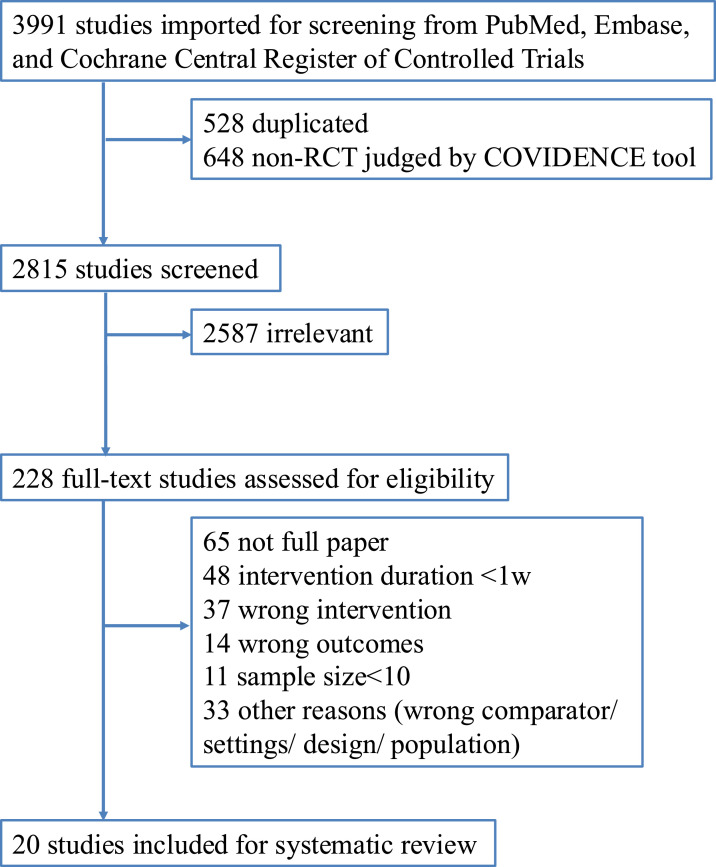
PRISMA flow chart.

Data were managed using COVIDENCE (https://www.covidence.org) throughout the review process. In PubMed, Embase and Cochrane Central Register of Controlled Trials (CENTRAL), we screened studies through November 4, 2024. Key search terms include "Hyperthermia, Induced" [MeSH], "Baths" [MeSH], "balneology" [MeSH], "bath*" [tiab] OR sauna* [tiab], and "heating" [tiab] to comprehensively capture passive heating interventions, and "Randomized Controlled Trial" [Publication Type], random* [tiab], and “trial” [tiab] to define study design (detailed search strategy is available in Appendix 1). A total of 1282, 1614, and 1095 articles were eligible from PubMed, Embase and CENTRAL, respectively. After removal of duplicates and non-RCT based on Cochrane RCT classifier [9], 2815 studies were retrieved and titles and abstracts were screened independently. Accordingly, 228 full-text articles were reviewed thoroughly for eligibility. A total of 20 RCTs were included in the systematic review, and data from each selected article were extracted by two independent reviewers and checked centrally for plausibility and integrity. The search algorithm is shown in detail in PRISMA Flowchart (Fig. 1). First author, journals, study designs, sample sizes, trial population, intervention design and duration, control design, measures of association, outcomes reported, and other trial characteristics were extracted. We reached out to the corresponding authors of any included papers that needed clarifications, and had a response from one author. Data extraction was independently conducted by two researchers and disagreements were resolved by unanimous consensus after discussion.

### 2.4 Data analysis

Revised Cochrane risk-of-bias tool for randomized trials was used to evaluate the risk of bias for RCTs [10]. The assessment included the following components: (1) risk of bias arising from the randomization process; (2) risk of bias due to deviation from the intended interventions; (3) missing outcome data; (4) risk of bias in measurement of the outcome; and (5) risk of bias in selection of the reported result.

Each outcome was analyzed separately. For outcomes reported in ≥3 RCTs, the absolute mean differences in the changes from baseline to follow-up comparing intervention and control arms were meta-analyzed. Given that many studies only reported means and SDs of outcomes at each timepoint, we calculated changes using the formula suggested by Cochrane handbook [11]. Briefly, for such RCTs, the means were calculated by subtracting pre-means from post-means. The SDs were calculated using the estimated correlation coefficient from the largest RCT that reported the SDs of changes. Given that the correlation coefficient for HbA1c outcome could not be calculated from a detailed study by Debray et al. due to small baseline SDs [8], we used correlation coefficient of 0.90 as a high correlation between pre- and post-intervention was expected. In sensitivity analysis, correlation coefficients of 0.70 and 0.80 were also examined.

Random-effects model meta-analyses were used to compute the weighted mean difference (WMD) in each outcome in a standardized unit and the 95 % confidence interval (CI). Existence of heterogeneity among effect sizes estimated by individual studies was described as the I^2^ index and the 95 % CI and tau. For results with high heterogeneity measures, we explored the sources of heterogeneity using stratification and meta-regression. The estimates were stratified by passive heating type (local or systemic; bathing or non-bathing), intervention length (<median 6 or ≥6 weeks), temperature (<median 42.8 °C or ≥42.8 °C), and underlying coronary risk or CVD (yes or no). Meta-regression was conducted for continuous covariates including heat temperature, total number of sessions, and intervention duration. Leave-one-out method was performed to better understand the uncertainty of the pooled results and examine the influence of an individual RCT estimate on the results [12]. We considered a study to be highly influential if its exclusion led to a substantial reduction in statistical heterogeneity or a change in the statistical significance of the overall pooled estimate. We examined Funnel plot asymmetry [13] and Egger’s test [14] to detect possible publication bias and small study effects. All analyses were conducted with R 4.4.0 (The R Foundation).

## 3 Results

Table 1 summarizes included studies in the present systematic review, with sample sizes ranging from 15 to 188 [8,[15], [16], [17], [18], [19], [20], [21], [22], [23], [24], [25], [26], [27], [28], [29], [30], [31], [32], [33]]. Controlled but non-randomized studies, such as those conducted by Brunt et al. [34], Ely et al. [35,36], and Kihara et al. [20], were not included in this review. Eight RCTs evaluated dry sauna [15,17,20,21,23,24,26,27], including six as Waon therapy for heart failure management conducted in Japan. Three RCTs evaluated Finish sauna with or without exercise [8,19,25]. Five RCTs assessed the effects of balneotherapy using warm to hot water [22,[30], [31], [32], [33]]. One balneotherapy RCTs for peripheral artery disease (PAD) reported outcomes separately for three groups according to the severity of arterial stenosis [32], and another RCT reported outcomes for obesity and obesity with hypertension separately [30]. Bikram hot yoga was evaluated in one RCT [18], and local heating interventions by three RCTs [16,28,29]. A study by Cheng et al. examined local heating intervention with or without exercise, compared with exercise only or no exercise, respectively [16]. Intervention durations spanned from 2 to 15 weeks, with one session consisting of 10- to 30-minute exposures to sauna or balneotherapy, 90 min to hot yoga, and 45 to 90 min to local heating. Included RCTs generally targeted older adults while three were based on young healthy adults [16,19,23].

**Table 1:** Included studies.

Author	Year	Country	Mean age	Women	Mean BMI	Disease	Sample size	Intervention	Follow-up	Temperature	Duration	Session N
Masuda [26]	2004	Japan	42 y	50 %	–	≥1 coronary risk	28	Dry sauna (Waon)	2 w	60 °C	15 min	10
Kihara [21]	2004	Japan	59 y	30 %	–	HF	30	Dry sauna (Waon)	2 w	60 °C	15 min	10
Miyata [27]	2008	Japan	64 y	34 %	–	HF	188	Dry sauna (Waon)	2 w	60 °C	15 min	10
Kuwahata [24]	2011	Japan	64 y	30 %	–	HF	54	Dry sauna (Waon)	4 w	60 °C	15 min	20
Fujita [17]	2011	Japan	65 y	18 %	–	HF	40	Dry sauna (Waon)	4 w	60 °C	15 min	20
Tei [15]	2016	Japan	66 y	39 %	21 kg/m^2^	HF	149	Dry sauna (Waon)	2 w	60 °C	15 min	10
Kunbootsri [23]	2013	Thailand	26 y	54 %	21 kg/m^2^	Allergic rhinitis	26	Dry sauna	6 w	80–90 °C	30 min	18
Lee [25]	2022	Finland	49 y	87 %	31 kg/m^2^	≥1 coronary risk	31	Finnish sauna + exercise	8 w	65 °C	15 min	24
Karolkiewicz [19]	2022	Poland	22 y	0 %	–	None	15	Finnish sauna + exercise	4 w	90 °C	30 min	12
Debray [8]	2023	Canada	62 y	20 %	–	Stable CAD	41	Finnish sauna	8 w	79 °C	20–30 min	32
Kudo [22]	2008	Japan	75 y	46 %	22 kg/m^2^	HF	26	Bathing	2 w	40 °C	10 min	14
Qiu [32]	2014	China	63 y	40 %	–	Diabetic PAD (stratified into 3 groups)	128	Bathing	4 w	39 °C	20 min	28
Roxburgh [33]	2023	New Zealand	66 y	47 %	32 kg/m^2^	Osteoarthritis	53	Bathing	12 w	40 °C	20–30 min	36
Oláh [30]	2011	Hungary	61 y	48 %	31 kg/m^2^	Obesity	44	Hajduszoboszlo spa	15 w	38 °C	30 min	75
Oláh [30]	2011	Hungary	66 y	73 %	39 kg/m^2^	Obesity, HTN	40	Hajduszoboszlo spa	15 w	38 °C	30 min	75
Oyama [31]	2013	Japan	69 y	50 %	23 kg/m^2^	HF	32	Hot springs	2 w	40 °C	10 min	10
Hunter [18]	2018	United States	48 y	28 %	29 kg/m^2^	None	40	Bikram yoga	12 w	40.5 °C	90 min	29–36
Monroe [28]	2020	United States	69 y	13 %	27 kg/m^2^	PAD	30	Water-circulating trousers (leg)	6 w	48 °C	90 min	18
Monroe [29]	2022	United States	66 y	27 %	28 kg/m^2^	PAD	30	Water-circulating trousers (leg)	12 w	43 °C	90 min	84
Cheng [16]	2024	Canada	23 y	50 %	24 kg/m^2^	None	30	Lower limb bathing (+exercise)	8 w	42.8 °C	45 min	24

Hajduszoboszlo spa is a large medicinal spa complex in Hungary famous for its unique, iodine-rich thermal water used for therapeutic bathing.

Bikram yoga is a specific sequence of 26 yoga postures and two breathing exercises performed in a room heated to ∼40 °C with ∼40 % humidity.

Waon therapy is a Japanese thermal therapy where the entire body is warmed in a far-infrared dry sauna uniformly maintained at 60 °C for 15 min.

Types of exercise evaluated were resistant and aerobic exercises (60min*3 session per week) in Lee’s study, bicycle ergometer in Karolkiewicz’s (60min*3 session per week) and Cheng’s (45min*3 session per week) studies.

Abbreviations: CAD, coronary artery disease, HF, heart failure, HTN, hypertension, PAD, peripheral artery disease.

### 3.1 Effects of passive heating on blood pressure

**Fig. 2:**
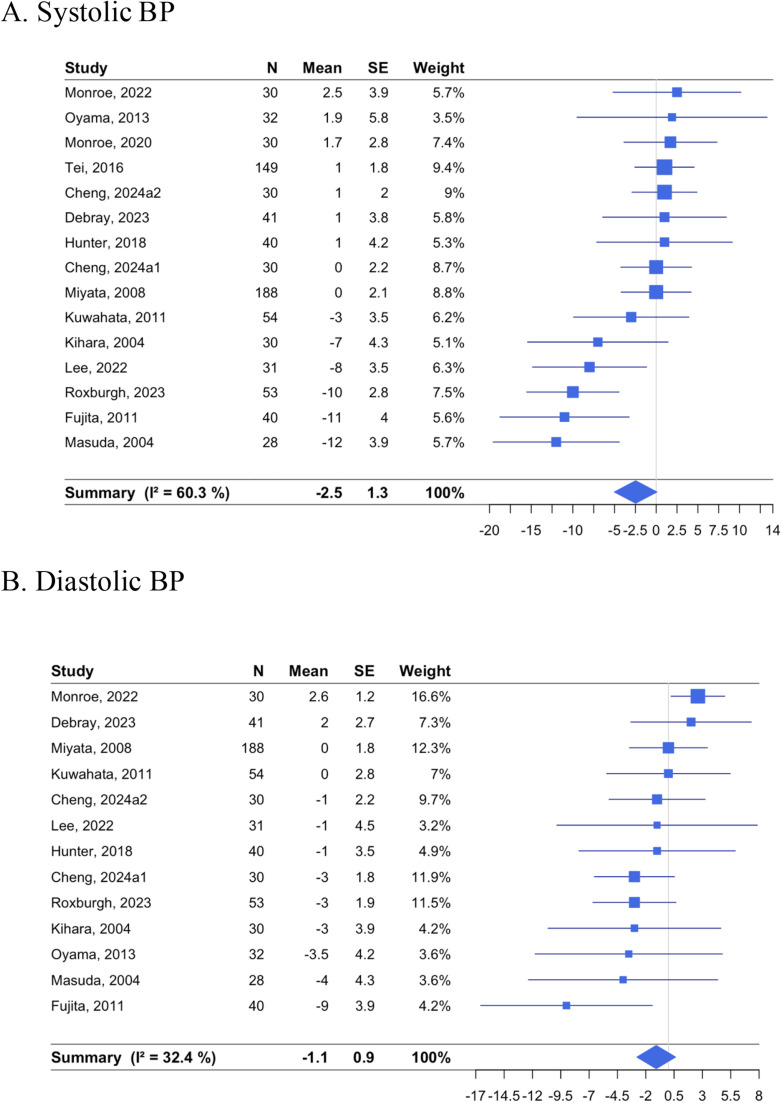
Effects of non-acute passive heating interventions on systolic and DBP. Forest plots showing the individual studies and the random-effect meta-analysis for the effects of non-acute passive heating interventions on changes in systolic (A) and diastolic (B) BP in mmHg.

Changes in SBP were evaluated in 15 RCTs (Fig. 2). The pooled estimate was −2.46 mmHg [95 % CI: −5.02, 0.10; *p* = 0.059] in the passive heating group compared with the control group, with an I^2^ of 60.3 % [30.2, 77.5]. In 13 RCTs evaluating the changes in DBP, the pooled estimate was −1.08 mmHg [ −2.79, 0.63; *p* = 0.21] with an I^2^ of 32.4 % [0.0, 65.1]. When stratified by intervention designs, local heating had no effects on changes in SBP (pooled estimate: 0.96 mmHg [−1.47, 3.38], I^2^ = 0 %), while systemic heating reduced SBP (−4.11 mmHg [−7.36, −0.86], I^2^ = 64.4 %), with significant between-group heterogeneity (*p* = 0.014; Table 2). There were also effects in populations with underlying coronary risk or CVD (−2.52 mmHg [−4.26, −0.79], *p* = 0.058 for between-group heterogeneity). Furthermore, intervention duration explained 24 % of the heterogeneity (*p* = 0.045; Supplemental Figure 1). Heat temperature or number of sessions did not explain any heterogeneity (0 %) in SBP. The heterogeneity for DBP was influenced by one RCT by Monroe et al. in which the sham had a greater reduction [29], and its exclusion led to a pooled estimate for DBP of −1.73 mmHg [−3.21, −0.26] with I^2^ of 0.0 % [0.0, 58.3] (Supplemental Figure 2). Types of exercise evaluated were resistant and aerobic exercises (60min*3 session per week) in Lee’s study [25], bicycle ergometer in Karolkiewicz’s (60min*3 session per week) [19] and Cheng’s (45min*3 session per week) [16] studies.

**Table 2:** Stratified meta-analyses to explore the sources of heterogeneity for systolic and diastolic blood pressure.

	N	Estimate [95 % CI]	I²	P-value for between-groups heterogeneity
***Changes in SBP***				
Local heating	4	0.96 [−1.47, 3.38]	0.0 %	0.014
Systemic heating	11	−4.11 [−7.36, −0.86]	64.4 %	
Bathing	4	−2.05 [−7.59, 3.49]	73.8 %	0.86
Non-bathing	11	−2.64 [−5.68, 0.41]	58.1 %	
Intervention length <6w	7	−3.93 [−8.14, 0.28]	65.3 %	0.36
Intervention length ≥6w	8	−1.40 [−4.73, 1.94]	60.1 %	
Mean temperature <42.8 °C	3	−3.27 [−11.4, 4.90]	69.7 %	0.79
Mean temperature ≥42.8 °C	12	−2.12 [−4.74, 0.51]	56.6 %	
No underlying coronary risk or CVD	3	0.59 [−2.12, 3.30]	0.0 %	0.058
Some underlying coronary risk or CVD	12	−2.52 [−4.26, −0.79]	65.2 %	
***Changes in DBP***				
Local heating	3	−0.22 [−3.72, 3.27]	72.5 %	0.51
Systemic heating	10	−1.55 [−3.30, 0.21]	0.0 %	
Bathing	4	−2.54 [−4.65, −0.43]	0.0 %	0.14
Non-bathing	9	−0.27 [−2.45, 1.91]	31.5 %	
Intervention length <6w	6	−2.09 [−4.82, 0.63]	8.4 %	0.37
Intervention length ≥6w	7	−0.49 [−2.71, 1.73]	44.7 %	
Mean temperature <42.8 °C	3	−2.68 [−5.70, 0.33]	0.0 %	0.29
Mean temperature ≥42.8 °C	10	−0.74 [−2.71, 1.23]	40.3 %	
No underlying coronary risk or CVD	3	−2.02 [−4.55, 0.51]	0.0 %	0.51
Some underlying coronary risk or CVD	10	−0.92 [−3.07, 1.24]	41.4 %	

Estimates [95 % CI], I^2^, and P-value for between-groups heterogeneity are based on random-effect meta-analysis.

### 3.2 Effects of passive heating on arterial stiffness

**Fig. 3:**
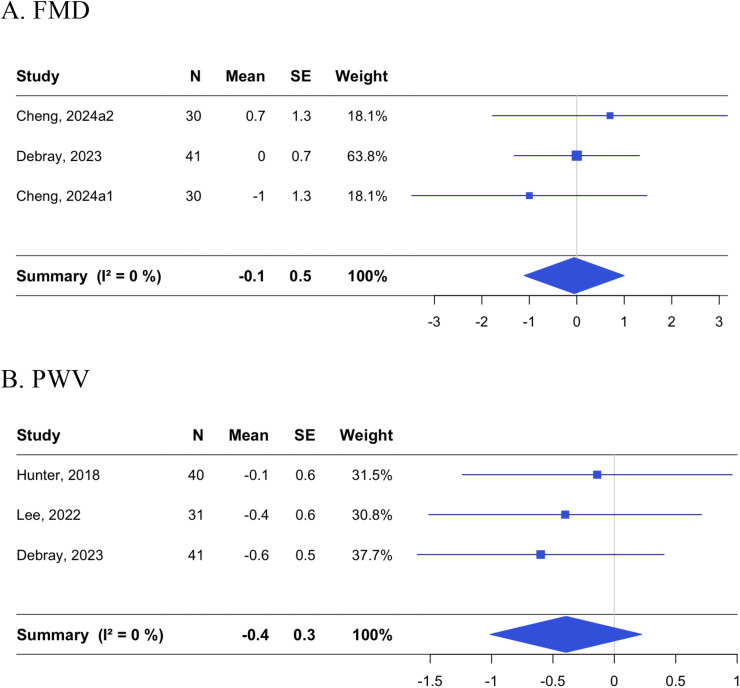
Effects of non-acute passive heating interventions on arterial stiffness measures. Forest plots showing the individual studies and the random-effect meta-analysis for the effects of non-acute passive heating interventions on changes in flow-mediated dilatation in % (A) and pulse wave velocity in m/s (B).

Changes in FMD and PWV were evaluated in 3 RCTs each, with one study reporting both outcomes [8] (Fig. 3). The pooled estimate for FMD was −0.055 % [−1.11, 1.00; *p* = 0.02] with I^2^ of 0.0 % [0.0, 89.6], and that for PWV was −0.39 m/s [−1.01, 0.22; *p* = 0.21] with I^2^ of 0.0 % [0.0, 89.6]. Lee et al. [25] additionally reported no significant effect of Finnish sauna combined with exercise on changes in augmentation index compared with exercise only (mean difference: −6.3 % [−14.8, 2.2]).

### 3.3 Effects of passive heating on heart rate and heart rate variability

**Fig. 4:**
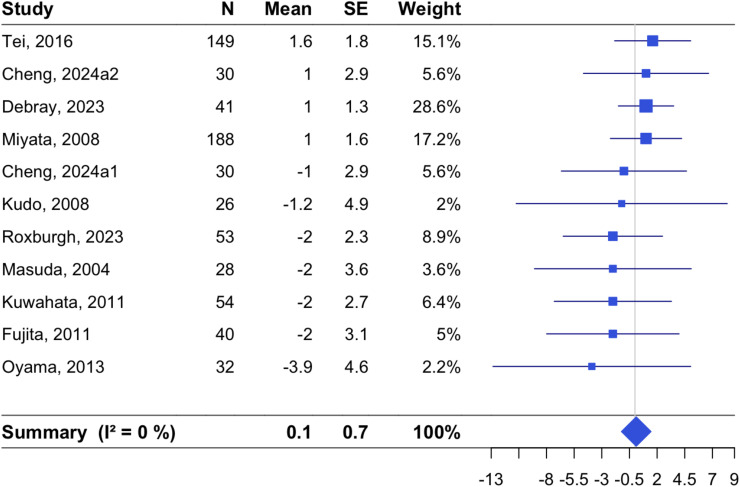
Effects of non-acute passive heating interventions on resting heart rate. Forest plots showing the individual studies and the random-effect meta-analysis for the effects of non-acute passive heating interventions on changes in resting heart rate in beats/minute.

Effects of passive heating on resting heart rate were reported in 11 RCTs (Fig. 4). The pooled estimate was 0.11 beats/minute [−1.22, 1.45; *p* = 0.87] with I^2^ of 0.0 % [0.0, 60.2]. No effect from smaller RCTs was observed for resting heart rate (Supplemental Figure 3). Effects on heart rate variability (HRV) were documented in only three RCTs; Kihara et al. showed a significant increase in standard deviation of NN interval (SDNN) by Waon therapy compared with control [21], while Roxburgh et al. reported no influence in several frequency domain HRV measures [33]. A study by Kunbootsri observed increased in HRV measured in both intervention and control groups [23].

### 3.4 Effects of passive heating on glucose metabolism

**Fig. 5:**
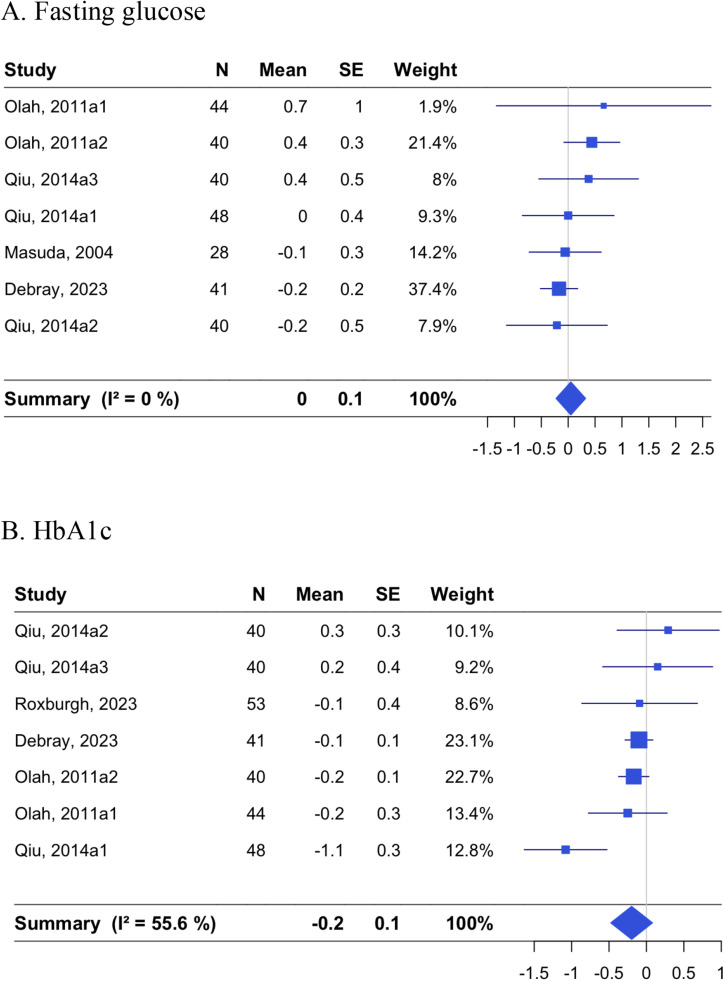
Effects of non-acute passive heating interventions on glucose metabolism. Forest plots showing the individual studies and the random-effect meta-analysis for the effects of non-acute passive heating interventions on changes in fasting glucose in mmol/L (A) and HbA1c in % (B).

Effects on fasting glucose and HbA1c were evaluated in 7 RCTs each (Fig. 5). The pooled estimate of changes in fasting glucose was 0.05 mmol/L [−0.23, 0.32; *p* = 0.73] with I^2^ of 0.0 % [0.0, 70.8], and for HbA1c changes were −0.20 % [−0.48, 0.08; *p* = 0.16] with I^2^ of 55.6 % [0.0, 80.9]. The heterogeneity for HbA1c result was explained by influence by Qiu et al. targeting patients with mild stenosis for diabetic lower extremity arterial disease [32], and its exclusion led to a pooled estimate of −0.11 % [−0.24, 0.01] with I^2^ of 0.0 % [0.0, 74.6] (Supplemental Figure 4). When a correlation coefficient of 0.8 or 0.7 for changes in HbA1c was used, the resulting pooled estimate was −0.15 % [−0.29, 0.00; *p* = 0.045] with I^2^ of 14.7 % [0.0, 58.4] and −0.14 % [−0.29, 0.02; *p* = 0.081] with I^2^ of 0.0 % [0.0, 70.8], respectively.

### 3.5 Effects of passive heating on lipid biomarkers

**Fig. 6:**
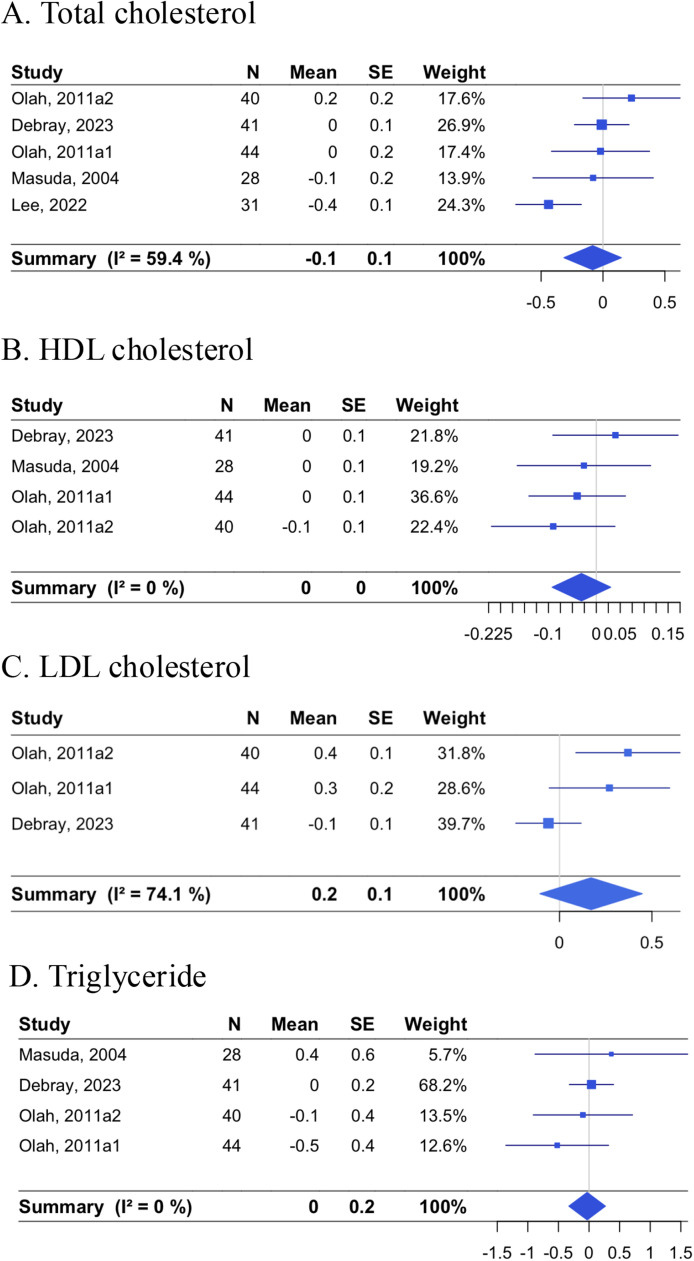
Effects of non-acute passive heating interventions on lipids. Forest plots showing the individual studies and the random-effect meta-analysis for the effects of non-acute passive heating interventions on changes in total cholesterol (A), HDL cholesterol (B), LDL cholesterol (C), and triglycerides (D), each in mmol/L.

Fig. 6 summarizes the effects of passive heating on changes in total, HDL, and LDL cholesterol, and triglycerides. The pooled estimates were −0.08 mmol/L [−0.31, 0.15; *p* = 0.48] for total cholesterol, −0.03 mmol/L [−0.09, 0.03; *p* = 0.32] for HDL cholesterol, 0.17 mmol/L [−0.11, 0.45; *p* = 0.23] for LDL cholesterol, and −0.03 mmol/L [−0.33, 0.27; *p* = 0.84] for triglycerides. An RCT by Lee et al. [25] influenced the pooled estimate for total cholesterol, and its omission led to a pooled estimate of 0.02 mmol/L [−0.14, 0.18] with I^2^ of 0.0 % [0.0, 84.7] (Supplemental Figure 5). The heterogeneity of the pooled estimate for LDL cholesterol (I^2^ of 74.1 %) was due to no effect and an increase in RCTs by Debray et al. and Oláh et al., respectively. In the Oláh study, although LDL cholesterol was reduced in both arms, the reduction was more pronounced in the control groups [30].

### 3.6 Effects of passive heating on inflammation

Only two studies reported effects of passive heating on changes in CRP. Oláh et al. reported no reduction in CRP by Hajduszoboszlo spa intervention among obese or hypertensive patients, while CRP was raised in the control group only among obese patients [30]. Karolkiewicz reported no effects of Finnish sauna combined with exercise in high sensitive CRP compared with exercise alone, but the population consisted of healthy, young individuals without elevated CRP [19].

### 3.7 Publication bias and risk of bias assessment

**Fig. 7:**
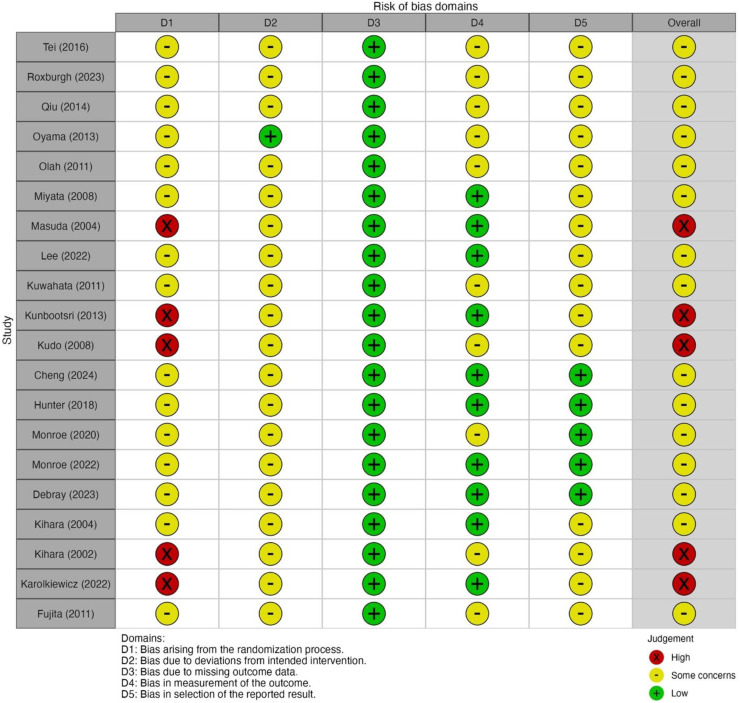
Summary of risk of bias assessment based on Revised Cochrane risk-of-bias tool for randomized trials.

Funnel plots for systolic and DBP outcomes are shown in Supplemental Figure 6, both looking asymmetrical, suggesting that either small study effects or publication bias exist for BP. The Egger’s test p-values were 0.12 and 0.041 for SBP and DBP, respectively. The p-value became not significant when a study by Monroe et al. [29] was omitted (*p* = 0.44), indicating that the small study effects explain the significant Egger’s test for DBP. Risk of bias assessment based on Cochrane risk of bias stool is visualized in Fig. 7, showing that most studies suffered from moderate to high risk of bias due to the non-blinded designs, imbalance between two arms due to small sample size, or lack of pre-registered protocols.

## 4 Discussion

Our systematic review and meta-analysis comprehensively summarized current evidence about the effects of passive heating on cardiometabolic and vascular health based on published RCTs. Overall, passive heating interventions did not lead to improvements in any cardiometabolic or vessel-based biomarkers. However, these findings are based on trials with small sample sizes with potential biases, and the pooled estimates had non-negligible heterogeneity and partly suffered from small study effects. When explored in detail, systemic but not local heating interventions and interventions targeting populations with coronary risk or CVD were most likely to reduce SBP. A reduction in DBP was observed with the omission of a single RCT in which the sham arm had a greater reduction. Despite no evidence to improve fasting glucose, passive heating might improve HbA1c. Finally, we found no effects for passive heating on arterial stiffness, resting heart rate or HRV measures, and lipids.

Hot water bathing and sauna bathing are popular practices embedded in some cultures like Japan and Finland. Epidemiological studies from both countries have shown strong associations between such passive heating behaviors and reduced cardiovascular risk [1,2], suggesting a role for passive heating behaviors for CVD prevention. With no large RCTs yet conducted, evidence is reliant on some smaller trials that have shown benefits from which past systematic reviews and meta-analyses have documented potential benefits on BP, arterial stiffness, and sleep [6,37]. However, the pooled estimates from previous meta-analyses had high heterogeneity, implying that there could be specific designs of a heating intervention or optimal targets to utilize the intervention for CVD prevention.

The present meta-analysis focused only on RCTs that evaluated changes of cardiometabolic or vascular outcomes in both intervention and control arms, and we explored the sources of heterogeneity in detail. The findings suggest that passive heating could lower SBP when applied to the whole body or targets populations with coronary risk or CVD. We found no strong evidence for other cardiometabolic or vascular effects, including arterial stiffness. However, our meta-analysis could not reliably show the effects due to potential biases, mainly stemming from trials with limited sample sizes. Funnel plots showed existence of either small study effects or publication bias, challenging the interpretation of the meta-analyses. Furthermore, in addition to statistical heterogeneity, there are non-ignorable clinical heterogeneities between studies, including types and durations of heating intervention, target population, and follow-up lengths, which could not be fully accounted for by stratification analyses. Therefore, the study is not conclusive in SBP and other findings, and a larger trial is warranted to clarify the clinical roles of passive heating interventions.

A few RCTs of passive heating are either planned or ongoing, including a crossover trial of hot water immersion on vascular health and BP among healthy older UK adults [38], a trial testing arm baths on BP among hypertensive German patients [39], and a planned hot water bathing intervention targeted to post-myocardial infarction patients [40]. While these studies will provide invaluable, targeted data on feasibility, safety, and preliminary efficacy in vulnerable groups, their small sample sizes (typically *N* = 20–50) will not be sufficient to definitively establish effects on CVD outcomes. Given an absence of conclusive trials, a larger-scale trial evaluating effects of daily whole-body heating interventions, potentially through hot water bathing, on changes in CVD biomarkers (BP in particular) over a year or longer, in adults with coronary risk would be highly warranted to advance our understandings of roles of passive heating in CVD prevention.

When designing such interventions, a nuanced understanding of the different heating modalities and their comfort and safety profiles is paramount. As reviewed, sauna (Finnish or far-infrared) and hot water bathing have been the most studied interventions, each differing in their capacity to raise core temperature, influence hemodynamics, and induce physiologic adaptations. Finnish saunas employ very high temperatures with low humidity and rely on shorter exposure times such as 5 to 20 min [41], while far-infrared (Waon) therapy achieves similar core heating at lower air temperatures, potentially offering better cardiovascular tolerance [42]. Hot water bathing, with its high thermal conductivity, can rapidly and uniformly raise core temperature at relatively mild water temperatures, and also has additional benefit from hydrostatic pressure [5,42], potentially suitable for daily usage by the general populations. Additionally, one study in younger adults showed that hot water bathing is associated with higher comfort than dry heat exposure in raising similar core temperature [43]. As for safety, saunas can increase risk of hypotension and arrythmia particularly combined with alcohol consumption [44]. Hot water bathing poses risks of unintentional drowning, particularly among the elderly who take long hot baths during the winter when the temperature of the dressing room/bathroom is low, and with alcohol drinking [[45], [46], [47]]. There are also potential risks of burns from excessively hot water and slips/falls upon entering or exiting the bath [48]. Future long-term RCTs must therefore incorporate robust safety protocols, including clear inclusion/exclusion criteria, participant education on hydration and alcohol avoidance, and careful monitoring for any adverse events to ensure both adherence and participant well-being.

There are several considerations impacting the interpretation of the present meta-analysis. First, most of the included trials had moderate to high risk of bias, largely due to the non-blinded designs, an imbalance between two arms resulted from the small sample sizes, or a lack of pre-registered protocols. However, the review minimized bias by strictly including trials with a control group, excluding several studies that evaluated only pre/post changes. Second, the review detected small study effects, high heterogeneity of the pooled estimates, and potential publication bias. We therefore conducted various analyses to account for them, including stratification, meta-regression, and leave-one-out methods, providing nuanced interpretations rather than just relying on the overall effects. Third, we imputed the means and SDs of the pre/post changes using an approach according to the Cochrane Handbook. Having individual patient data would have allowed for greater precision. Fourth, measured BP is a highly variable outcome that could potentially fluctuate from time to time, whereas most previous trials did not evaluate BP at multiple time points. A larger RCT with longer duration and multiple follow-ups would be highly informative by showing BP projections over time, accounting for the issue.

## 5 Conclusion

This systematic review and meta-analysis of smaller RCTs did not find overall improvements in cardiometabolic or vascular biomarkers following passive heating interventions, likely due to moderate RCT quality and high heterogeneity. When evaluated in detail, a potential reduction in SBP was observed in systemic heating interventions and populations with coronary risk or CVD, while this observation was based on small trials and not conclusive. Despite the promising epidemiological associations observed in cultures with regular passive heating practices, current RCT evidence does not conclusively support the effectiveness of passive heating for cardiometabolic health improvement. Larger, well-designed RCTs are needed to clarify the potential role of passive heating as a feasible non-pharmacological intervention for CVD prevention.

## CRediT authorship contribution statement

**Rikuta Hamaya:** Writing – original draft, Visualization, Project administration, Methodology, Investigation, Formal analysis, Conceptualization. **Yuki Joyama:** Writing – review & editing, Methodology, Investigation, Formal analysis, Data curation. **Tomohiro Miyata:** Writing – review & editing, Investigation, Data curation. **Shun-ichiro Fuse:** Writing – review & editing, Investigation, Data curation. **Naho Yamane:** Writing – review & editing, Investigation, Data curation. **Natsuki Maruyama:** Writing – review & editing, Investigation, Data curation. **Hirofumi Kanazawa:** Writing – review & editing, Investigation, Data curation. **Koki Morishita:** Writing – review & editing, Investigation, Data curation. **Howard D. Sesso:** Writing – review & editing, Supervision, Project administration, Investigation.

## Declaration of competing interest

The authors declare the following financial interests/personal relationships which may be considered as potential competing interests: Rikuta Hamaya is an owner of Everyone Cohort Inc. Howard Sesso reported receiving investigator-initiated grants from Pure Encapsulations and the American Pistachio Growers, and honoraria and/or travel for lectures from the Council for Responsible Nutrition, BASF, Haleon, and NIH. No other authors reported any conflicts of interests for this study. If there are other authors, they declare that they have no known competing financial interests or personal relationships that could have appeared to influence the work reported in this paper.

## Appendix Supplementary materials

Image, application 1
