# Supplementary material for: Non-acute effects of passive heating interventions on cardiometabolic risk and vascular health: systematic review and meta-analysis of randomized controlled trials
Source: Am J Prev Cardiol. 2025 Aug 17;23:101082. doi: 10.1016/j.ajpc.2025.101082 (PMC12490526; doi:10.1016/j.ajpc.2025.101082)
Supplement: Supplementary file 1 [file mmc1.docx]

Supplemental data

*Hamaya et al. Non-acute effects of passive heating interventions on cardiometabolic risk biomarkers: systematic review and meta-analysis of randomized controlled trials*

**Appendix:** Search strategies

**Supplemental Figure 1:** Meta-regression for changes in SBP by intervention designs

**Supplemental Figure 2:** Leave-one-out methods for systolic and DBP outcomes

**Supplemental Figure 3:** Leave-one-out methods for resting heart rate outcome

**Supplemental Figure 4:** Leave-one-out methods for glucose metabolism outcomes

**Supplemental Figure 5:** Leave-one-out methods for lipids

**Supplemental Figure 6:** Publication bias for BP outcomes

**Appendix:** Search strategies

**Pubmed**

(

("Hyperthermia, Induced"[MeSH] OR "Baths"[MeSH] OR "hot springs"[MeSH] OR "balneology"[MeSH] OR "hot spring"[tiab:~3] OR "hot springs"[tiab:~3] OR "hot immersion"[tiab:~5] OR "warm immersion"[tiab:~5] OR "bath*"[tiab] OR sauna*[tiab] OR "hot tub*"[tiab] OR "passive heat stress"[tiab] OR "heat acclimation"[tiab] OR "heat training"[tiab] OR "heat therap*"[tiab] OR "heat exposure"[tiab] OR "heating"[tiab] OR "waon"[tiab] OR "balneo*"[tiab] OR "hot stimul*"[tiab])

)

AND

(

("Blood Pressure"[MeSH] OR "Glycated Hemoglobin"[MeSH] OR "Insulin"[MeSH] OR "Cholesterol"[MeSH:noexp] OR "Cholesterol, LDL"[MeSH] OR "Cholesterol, HDL"[MeSH] OR

"Triglycerides"[MeSH] OR "Interleukin-6"[MeSH] OR "Tumor Necrosis Factor-alpha"[MeSH] OR "C-Reactive Protein"[MeSH] OR "blood pressure"[tiab] OR hypertension[tiab] OR "HbA1c"[tiab] OR "glucose"[tiab] OR "insulin"[tiab] OR diabetes[tiab] OR

"cholesterol"[tiab] OR "lipid*"[tiab] OR "IL-6"[tiab] OR il6[tiab] OR interleukin-6[tiab] OR "TNF "[tiab] OR "Tumor Necrosis Factor"[tiab] OR "C-Reactive Protein"[tiab] OR "CRP"[tiab] OR "inflammat*"[tiab] OR cardiometabol*[tiab])

OR

("Vasodilation"[MeSH] OR "Endothelium, Vascular"[Mesh] OR "Heart Failure"[Mesh] OR “flow mediated dilatation”[tiab] OR “flow-mediated dilatation”[tiab] OR “FMD”[tiab] OR “pulse wave velocity”[tiab] OR “pulse-wave velocity”[tiab] OR “PWV”[tiab] OR “augmentation index”[tiab] OR “pulse wave analys*”[tiab] OR “PWA”[tiab] OR “pulse pressure*”[tiab] OR "vascular function*"[tiab] OR "vascular health"[tiab] OR “endothelial function*”[tiab] OR “microvascular”[tiab] OR “heart failure”[tiab] OR “arterial pressure”[tiab] OR "vascular pressure”[tiab] OR“arterial stiffness”[tiab] OR “vascular stiffness”[tiab] OR "carotid artery function"[tiab] OR "artery compliance*"[tiab] OR "arterial compliance*"[tiab] OR "artery distensibilit*"[tiab] OR "arterial distensibilit*"[tiab] OR “heart rate variabilit*”[tiab] OR “HRV”[tiab] OR “autonomic nerv*”[tiab] OR “baroreflex sensitivit*”[tiab] OR “BRS”[tiab] OR “blood pressure variabilit*”[tiab])

)

AND

("Randomized Controlled Trial"[Publication Type] OR random*[tiab] OR “trial”[tiab])

AND English[language]

NOT

("Animals"[Mesh] NOT "Humans"[Mesh])

**Embase**

**(**

('induced hyperthermia'/exp OR 'bath'/exp OR ‘balneology‘/exp) OR

((hot NEAR/3 springs) OR (('hot water' OR 'warm water') NEAR/5 immersion) OR 'bath*' OR sauna* OR 'hot tub*' OR 'passive heat stress' OR 'heat acclimation' OR 'heat training' OR 'heat therap*' OR 'heat exposure' OR 'heating' OR 'waon' OR 'balneo*' OR 'hot stimul*'):ab,ti,kw

**)**

AND

(

(

('glycated hemoglobin'/exp OR 'insulin'/exp OR 'interleukin 6'/exp OR 'c-reactive protein'/exp OR 'tumor necrosis factor alpha'/exp) OR

('blood pressure' OR hypertension OR 'HbA1c' OR 'glucose' OR 'insulin' OR diabetes OR

'cholesterol' OR 'lipid*' OR 'IL-6' OR il6 OR interleukin-6 OR 'TNF ' OR 'Tumor Necrosis Factor' OR 'C-Reactive Protein' OR 'CRP' OR 'inflammat*' OR cardiometabol*):ab,ti,kw

)

OR

('flow mediated dilatation' OR 'flow-mediated dilatation' OR 'FMD' OR 'pulse wave velocity' OR 'pulse-wave velocity' OR 'PWV' OR 'augmentation index' OR 'pulse wave analys*' OR 'PWA' OR 'pulse pressure*' OR 'vascular function*' OR 'vascular health' OR 'endothelial function*' OR 'microvascular' OR 'heart failure' OR 'arterial pressure' OR 'vascular pressure' OR 'arterial stiffness' OR 'vascular stiffness' OR 'carotid artery function' OR 'artery compliance*' OR 'arterial compliance*' OR 'artery distensibilit*' OR 'arterial distensibilit*' OR 'heart rate variabilit*' OR 'HRV' OR 'autonomic nerv*' OR 'baroreflex sensitivit*' OR 'BRS' OR 'blood pressure variabilit*'):ab,ti,kw

)

AND

('randomized controlled trial'/exp OR 'random*':ti,ab,kw OR 'trial':ti,ab,kw)

AND

[english]/lim

AND

[embase]/lim

NOT

('animal'/exp NOT 'human'/exp)

**Cochrane Central Register of Controlled Trials:**

(((hot NEAR/3 springs) OR (hot NEAR/5 immersion) OR (warm NEAR/5 immersion) OR bath* OR sauna* OR (hot NEXT tub*) OR (heat NEXT stress) OR (heat NEXT acclimation) OR (heat NEXT training) OR (heat NEXT therap*) OR (heat NEXT exposure) OR heating OR waon OR balneo* OR (hot NEXT stimul*))

AND

((flow-mediated NEXT dilatation) OR (flow NEXT mediated NEXT dilatation) OR fmd OR (pulse NEXT wave NEXT velocity) OR (pulse-wave NEXT velocity) OR PWV OR (augmentation NEXT index) OR (pulse NEXT wave NEXT analys*) OR PWA OR (pulse NEXT pressure*) OR (vascular NEXT function*) OR (vascular NEXT health) OR (endothelial NEXT function*) OR microvascular OR (heart NEXT failure) OR (arterial NEXT pressure) OR (vascular NEXT pressure) OR (arterial NEXT stiffness) OR (vascular NEXT stiffness) OR (carotid NEXT artery NEXT function) OR (artery NEXT compliance*) OR (arterial NEXT compliance*) OR (artery NEXT distensibilit*) OR (arterial NEXT distensibilit*) OR (heart NEXT rate NEXT variabilit*) OR HRV OR (autonomic NEXT nerv*) OR (baroreflex NEXT sensitivit*) OR BRS OR (blood NEXT pressure NEXT variabilit*) OR (blood NEXT pressure) OR hypertension OR HbA1c OR glucose OR insulin OR diabetes OR cholesterol OR lipid* OR IL-6 OR il6 OR interleukin-6 OR TNF OR (Tumor NEXT Necrosis NEXT Factor) OR (C-Reactive NEXT Protein) OR CRP OR inflammat* OR cardiometabol*))

NOT

*((Embase):an OR (Pubmed):an )*

**Supplemental Figure 1:** Meta-regression for changes in SBP by intervention designs


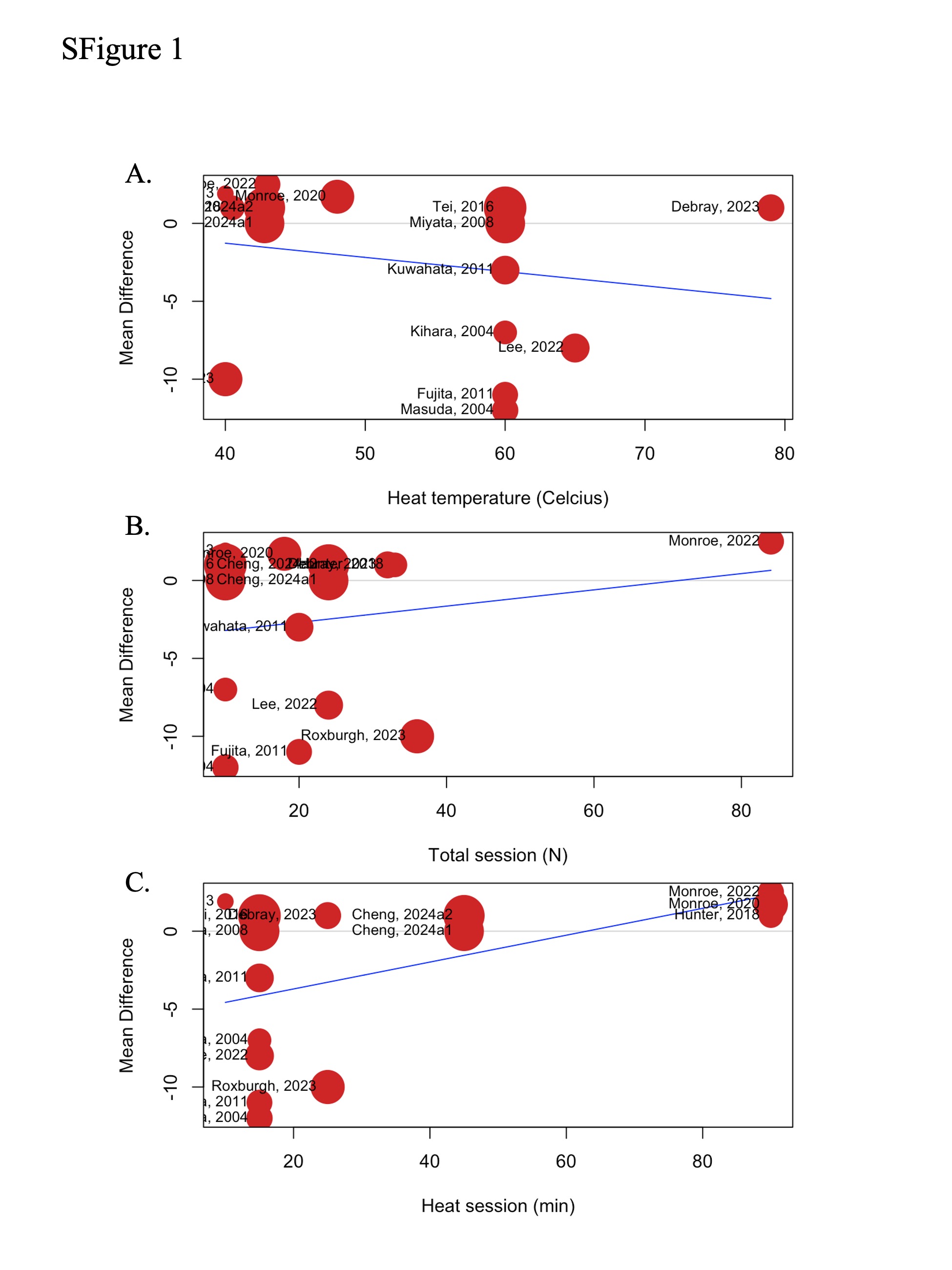


Meta-regression results for SBP outcome by heat temperature (A), total number of sessions (B), and duration of single heat session (C). Each dot represents an individual RCT and the radium indicates the weights.

**Supplemental Figure 2:** Leave-one-out methods for systolic and DBP outcomes


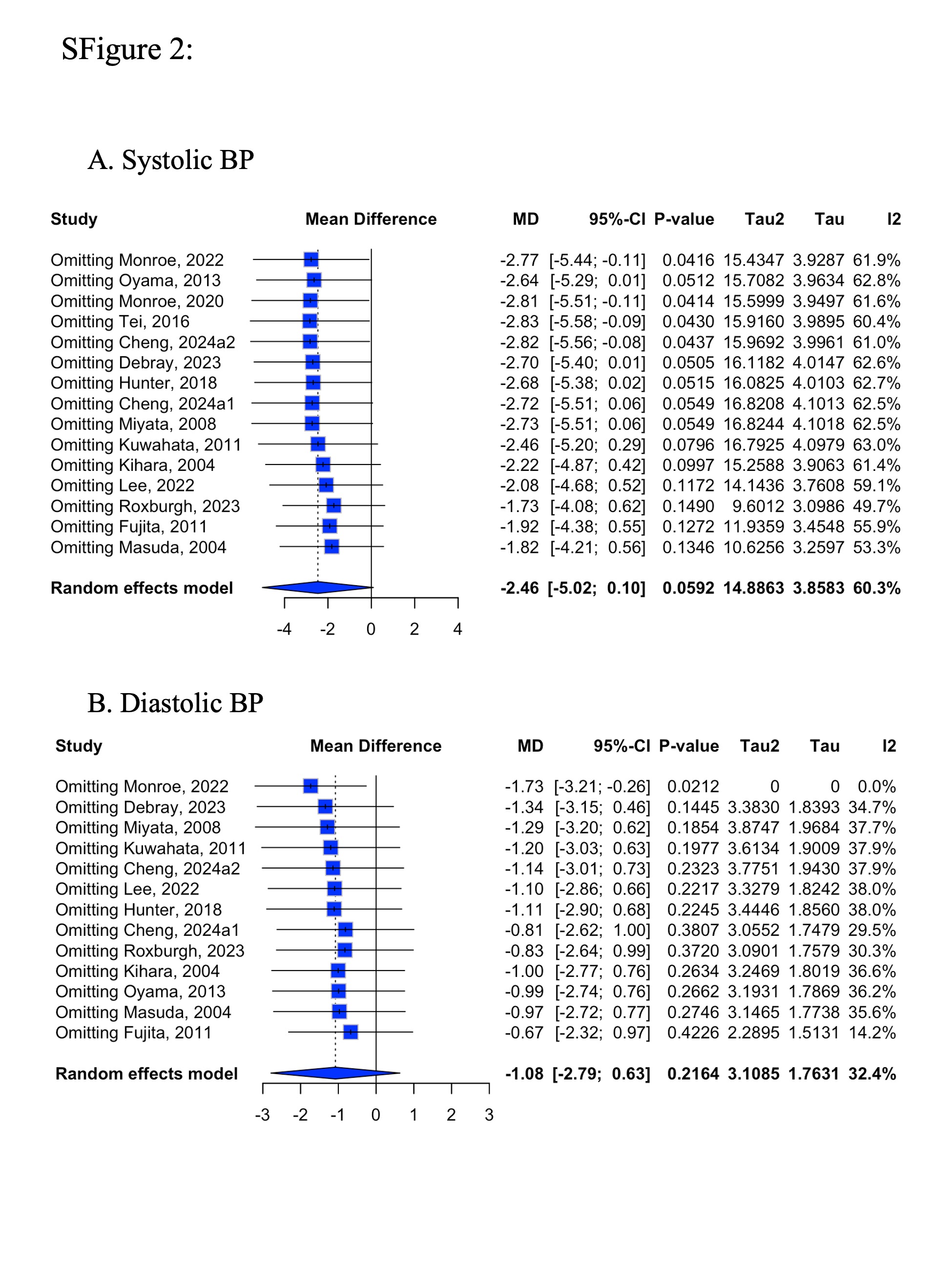


Results of leave-one-out methods for systolic (A) and DBP (B). Each row indicates the random-effect meta-analysis results when one of the included studies is separately omitted. No major changes were observed for SBP, whereas meta-analysis estimates and heterogeneity measures were changed for DBP when a study by Monroe was omitted, indicating a small study effect.

**Supplemental Figure 3:** Leave-one-out methods for resting heart rate outcome


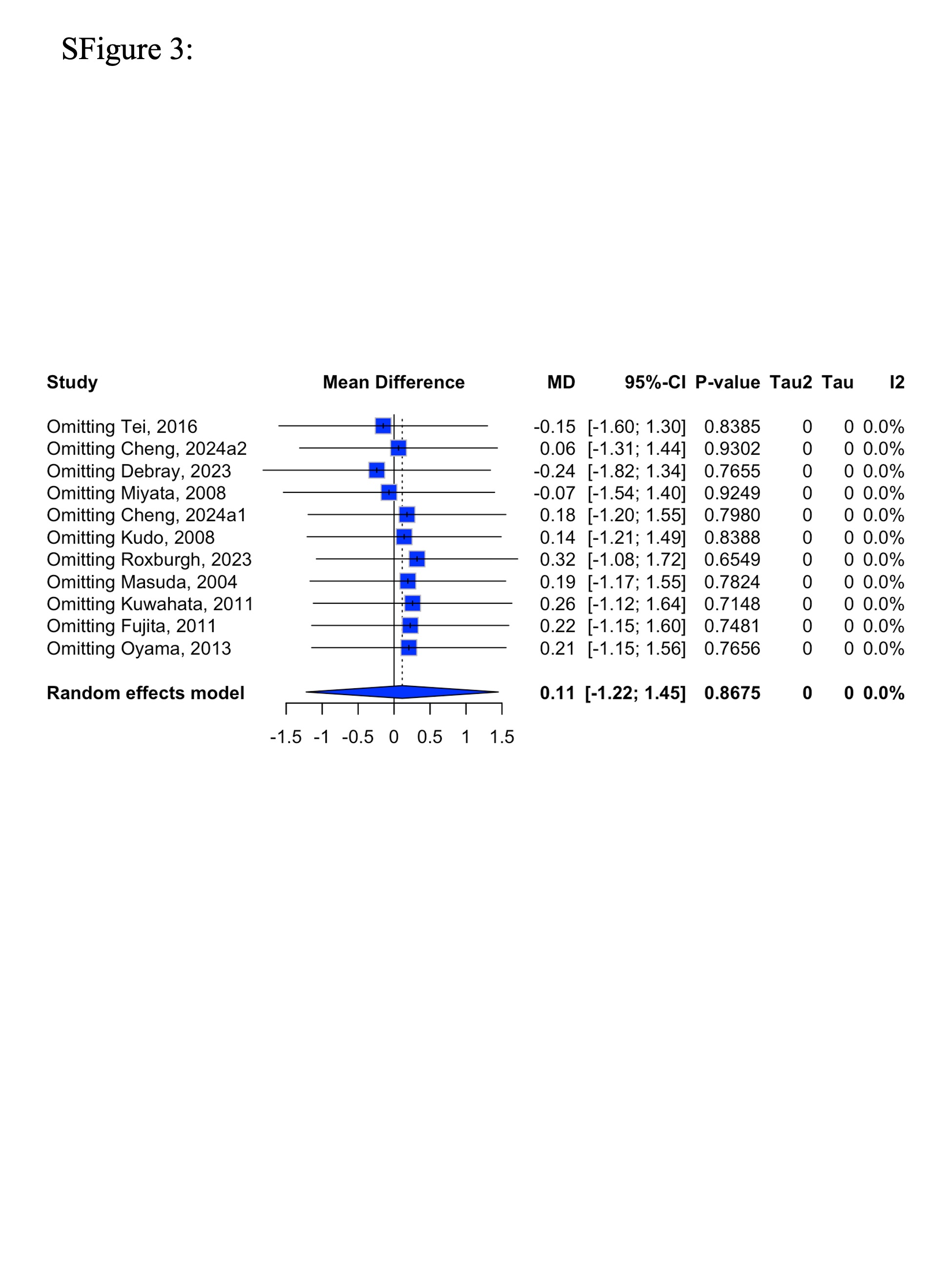


Results of leave-one-out methods for resting heart rate. Each row indicates the random-effect meta-analysis results when one of the included studies is separately omitted. No apparent small study effect was observed.

**Supplemental Figure 4:** Leave-one-out methods for glucose metabolism outcomes


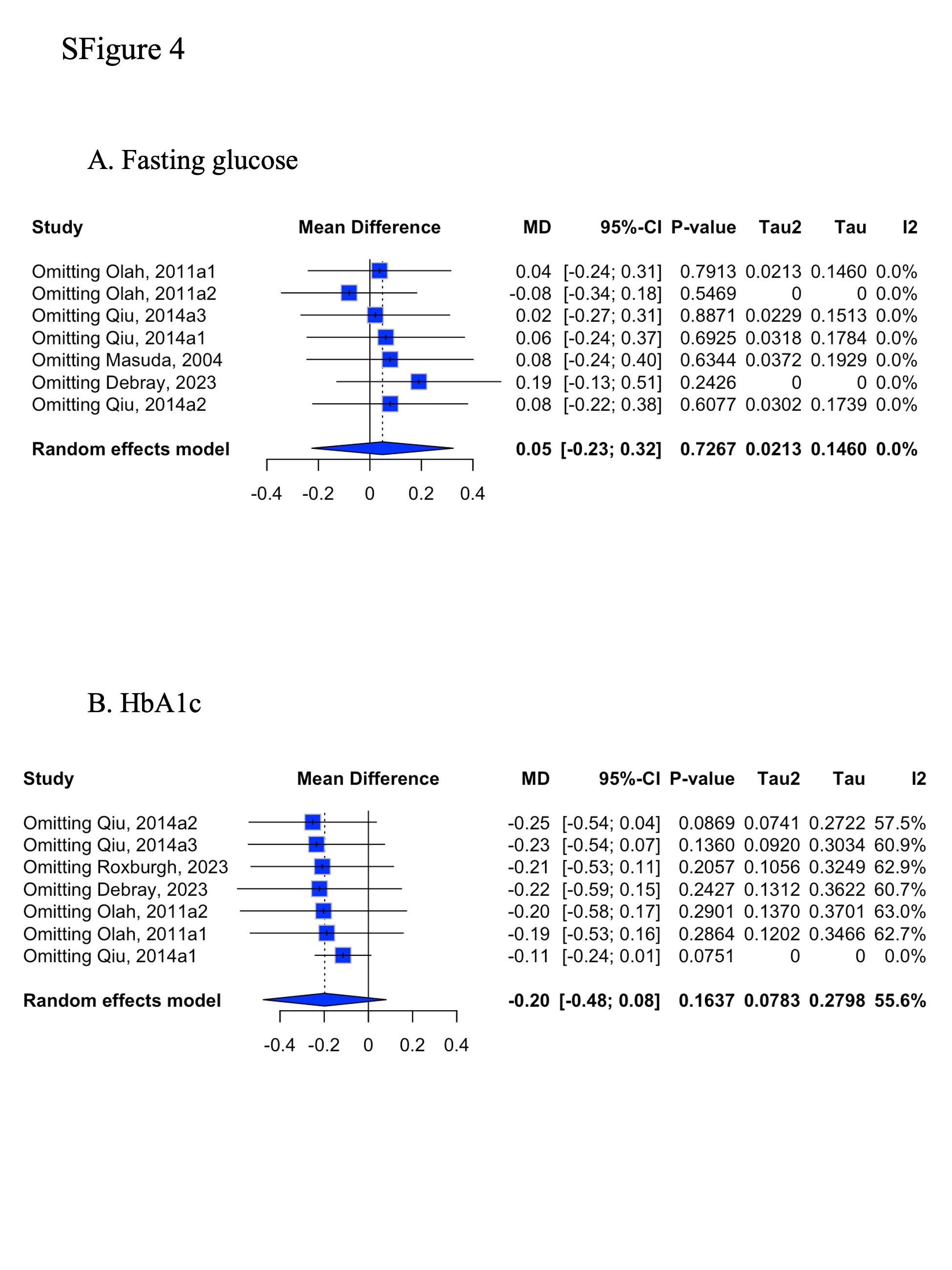


Results of leave-one-out methods for fasting glucose (A) and HbA1c (B). Each row indicates the random-effect meta-analysis results when one of the included studies is separately omitted. No major changes were observed for fasting glucose, whereas meta-analysis estimates and heterogeneity measures were drastically changed for HbA1c when a population of the study by Qiu (Qiu, 2014a1) was omitted, indicating a small study effect.

**Supplemental Figure 5:** Leave-one-out methods for lipids


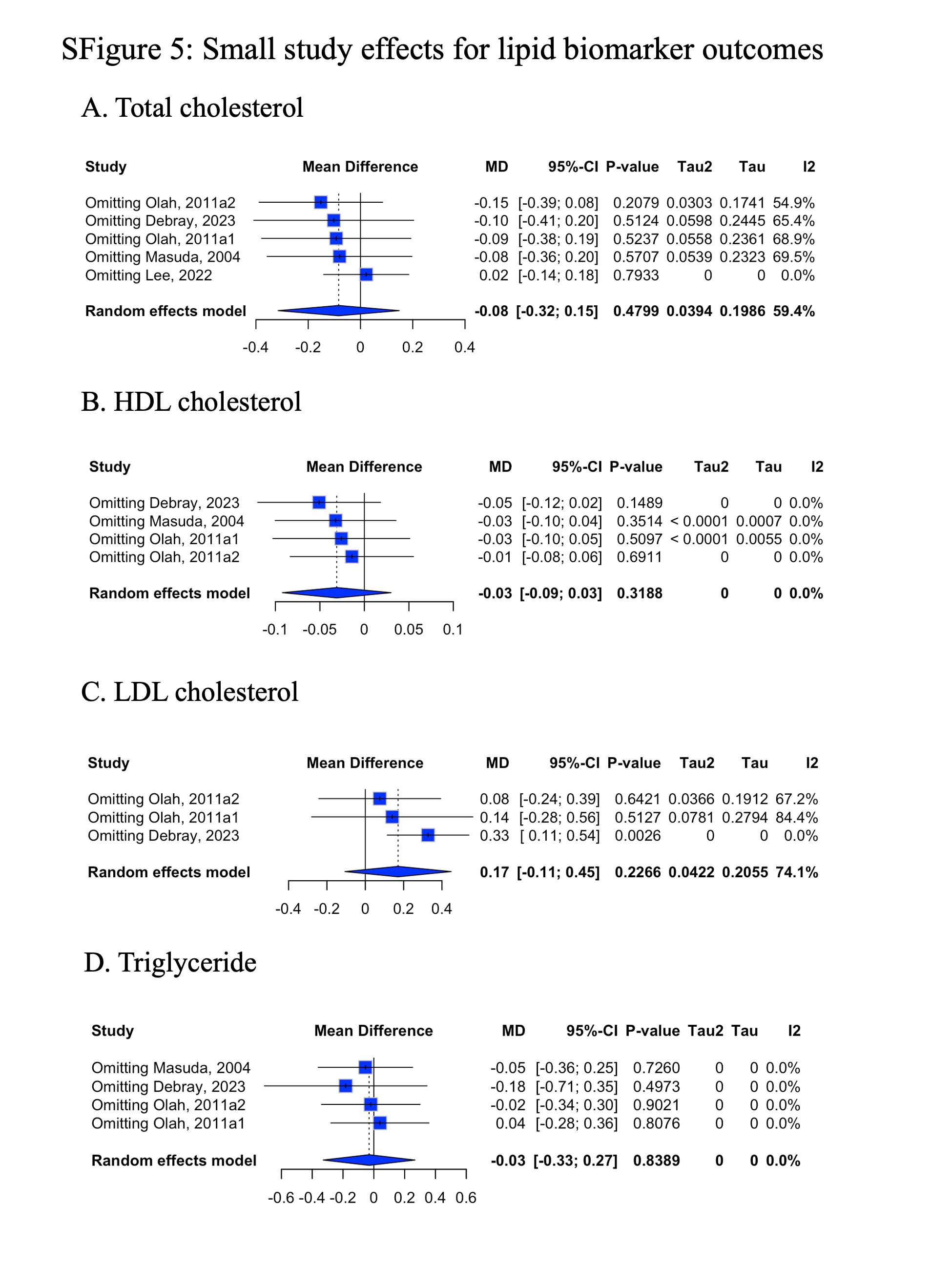


Results of leave-one-out methods for total cholesterol (A), HDL cholesterol (B), LDL cholesterol (C), and triglycerides (D). Each row indicates the random-effect meta-analysis results when one of the included studies is separately omitted. Existence of small study effects were suggested for total cholesterol and LDL cholesterol.

**Supplemental Figure 6:** Publication bias for BP outcomes


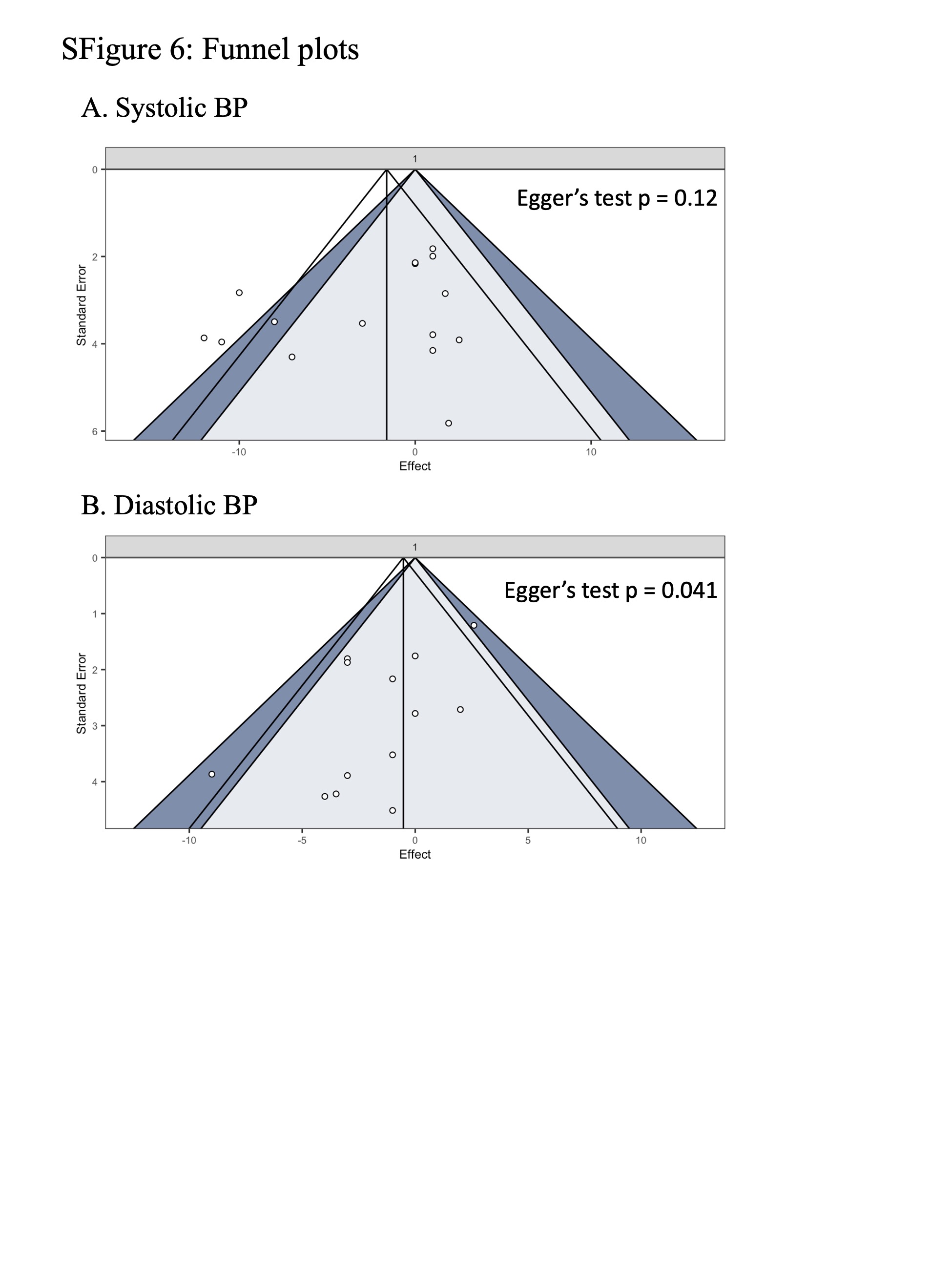


Results of Funnel plot and Egger’s test for systolic (A) and DBP (B). Each funnel plot shows asymmetry, suggestive of an existence of small study effect or publication bias. Significant Egger’s test for DBP indicates a small study effect.
